# Epidermodysplasie verruciforme: à propos d’un cas

**DOI:** 10.11604/pamj.2018.30.78.16058

**Published:** 2018-05-29

**Authors:** Fatima-Zahra Agharbi

**Affiliations:** 1 Hôpital Civil Tétouan, Tétouan, Maroc

**Keywords:** Epidermodysplasie, verruciforme, HPV, carcinomes, Epidermodysplasia, verruciformis, HPV, carcinomas

## Abstract

Epidermodysplasia verruciformis, also known as Lewandowsky-Lutz syndrome or tree man disease is a rare genetic skin disorder. It is characterized by abnormal susceptibility of the skin coating to human papillomaviruses (HPVs). It commonly affects people between the ages of 4 and 8, most often before the age of 20 years but it may exceptionally occur later. It is characterized by the appearance of scaly macules and sometimes exuberant pseudotumoral papules, mainly on hands and feet. HPV 5 and HPV-8 have been characterized in these lesions, viruses that are found in about 80% of the normal population. Other HPV types can sometimes be identified. Felix Lewandowskyand-Wilhelm Lutz provided the first clinical description of this disorder. The most frequently observed skin lesion is a macular rash similar to that of pityriasis versicolor associated with verrucous scaly papules. The risk of malignant transformation to squamous cell carcinoma is high. Several treatments have been tried (retinoids, interferon, cimetidine) with little or non-repeatable success. Sun protection, assiduous clinical monitoring and early excision of any lesion undergoing malignang transformation are essential. We report the case of a 30 year old man presenting with multiple verrucous lesions on limbs and trunk occurred at the age of 4 years associated with pityriasis versicolor-like lesions. Clinical examination didn't show suspicious lesions and retinoid therapy was started.

## Image en médecine

L'épidermodysplasie verruciforme, également appelée **syndrome de Lutz-Lewandowsky** ou la maladie de l'homme-arbre est une affection cutanée rare d'origine génétique. Elle se caractérise par une sensibilité anormale du revêtement cutané aux papillomavirus. L'affection commence habituellement entre 4 et 8 ans, le plus souvent avant l'âge de 20 ans mais peut exceptionnellement apparaitre plus tardivement. Elle se traduit par l'apparition de macules squameuses et de papules d'évolution parfois exubérante, pseudo-tumorale, essentiellement au niveau des mains et des pieds. Dans ces lésions, on retrouve des papillomavirus de type 5 et 8, virus que l'on retrouve chez 80% des sujets d'une population normale asymptomatique. D'autres types de papillomavirus peuvent être parfois identifiés. Felix Lewandowsky et Wilhelm Lutz ont fait la première description clinique de cette affection. La lésion cutanée la plus fréquemment observée est une éruption maculaire proche de celle connue dans le pityriasis versicolor; s'y associent des papules d'allure verruqueuses squameuses. Le risque de transformation particulièrement en carcinome épidermoide est important. Plusieurs traitements ont été essayés (rétinoïdes, interféron, cimétidine) avec des succès peu ou pas reproductibles. Le plus important est la protection solaire, la surveillance clinique assidue et l'excision rapide de toute lésion en voie de dégénérescence carcinomateuse. Nous rapportons l'observation d'un homme de 30 ans qui consulte pour des lésions verruqueuses multiples des membres et tronc remontant à l'âge de 4 ans associés à des lésions pityriasis versicolor like. L'examen ne trouvait pas de lésions suspectes et un traitement à base de rétinoides a été démarré.

**Figure 1:**
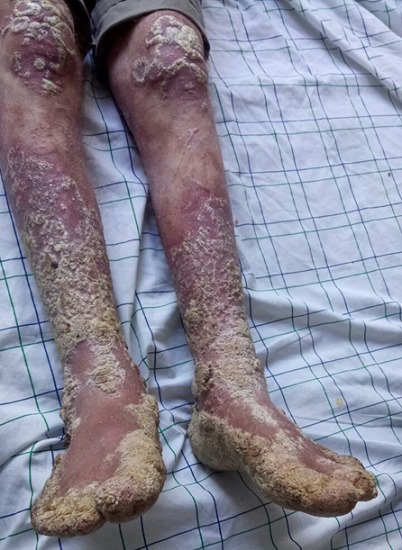
Plaques verruqueuses de membres inférieurs

